# The implementation of scribing within a medical school’s pre-clinical curriculum: pilot study

**DOI:** 10.1186/s12909-022-03379-7

**Published:** 2022-04-25

**Authors:** Vanessa Palomares, Arpan Patel, Ellen Wagner, Elisa McCarthy, William Adams, Matthew Fitz

**Affiliations:** 1 Loyola University Chicago Stritch School of Medicine, Maywood, IL USA; 2 Department of Pediatrics, University of Chicago, Chicago, IL USA; 3 Department of Pediatrics, University of Utah School of Medicine, Salt Lake City, UT USA; 4 Department of Medical Education, Loyola University Chicago Stritch School of Medicine, Maywood, IL USA; 5 Department of Internal Medicine, Loyola University Chicago Stritch School of Medicine, Maywood, IL USA

**Keywords:** Scribe, Electronic medical record, Preclinical medical education, Clinical developments

## Abstract

**Background:**

Medical students matriculating from their preclinical curriculum into clinical clerkships face a significant learning curve when using an electronic medical record (EMR) system for clinical documentation. With the trend toward reduction in preclinical medical education, students now have fewer opportunities to optimize their note-writing and overall clinical skills before transitioning to patient-care settings.

**Methods:**

This study sought to investigate how a structured medical scribing program in an outpatient clinic helps bridge the gap between traditional preclinical and clinical curricula in medical education. A small cohort of medical students were trained in medical scribing within our institutions’ existing preclinical preceptorship program. We surveyed students, preceptors, and patients during the project to better understand confidence around documentation, the EMR, and the impact of the scribing program on workflow efficiency and patient satisfaction.

**Results:**

There was no significant difference between the scribe and non- scribe students in their confidence documenting a patient encounter or navigating EMR (all *p* > .05). Our study demonstrated that preceptors for scribe students reported a significant decrease in documentation time compared to non-scribes (*M*diff = − 5.75, *p* = .02), with no negative impact on patient satisfaction.

**Conclusions:**

Medical scribing can be a tool to further develop medical trainees in clinical documentation and help prepare them for the responsibilities during clinical years. When summing the per encounter time savings over the course of a half or full clinic day, scribing can return a significant amount of time back to preceptors. The time saved by the preceptor needs to be further investigated to determine if the time can lend itself towards better patient care, student-specific feedback, focused teaching, or even mentoring.

## Background

Recent changes in the United States include a trend toward shortened preclinical time prior to entering the traditional clinical years [1]. The incorporation of a flipped-classroom model, another significant change to medical education in which students are exposed to new material outside the classroom, ideally replaces hours of traditional lectures with condensed sessions in which students are asked to actively apply material with higher level cognitive work [2]. However, students have raised concerns over the time intensiveness of outside preparation without the ability to ask questions in real time or access faculty support with overly complex subject matter [2]. Furthermore, depending on its structure the flipped classroom sessions may require more faculty resources for small group learning and can be negatively impacted by inadequate levels of students’ preparedness [2]. A now growing student population with a truncated preclinical time and less in-person didactic education faces a steep learning curve entering clinical years, balancing responsibilities to patient care alongside sufficient clinical documentation.

The implementation of scribing in medical education may address the clinical documentation learning by allowing the scribe to actively participate by charting the physician-patient encounter instead of passively listening. The traditional role of medical scribes includes observing and documenting medical encounters in real-time. Prior to initiating their work, scribes are trained in medical record navigation, documentation techniques, and medical terminology.

Numerous studies show scribes improving patient experience and satisfaction, clinical satisfaction, and increasing productivity [3–5]. This productivity is realized as decreased documentation time and increased work productivity as measured by clinical revenue [4, 6, 7]. Scribes in the emergency department as well as medical students in their clinical years have stated scribing is an overall positive experience that enhances engagement, physician feedback, and fosters a more team-based culture [5, 8]. Furthermore third- and fourth-year medical students functioning as scribes had greater teaching focus, contributions to a teamwork culture, and service as an EHR resource [8]. Scribes who were applying to medical school reported that they learned medical terminology, observed communication between the doctor and patient, and understood the practice of medicine in a clinical setting [9]. Given the clinical training as well as intensive exposure to medical decision making involved, it is easy to envision how former scribes may have an advantage compared to peers in terms of navigating the medical system. All of the reported literature analyzed the impact on third- and fourth-year medical students or individuals prior to medical school. There is little investigation on preclinical (i.e., M1 or M2) incorporation of scribing in medical education. To our knowledge, no studies have addressed the utility of medical scribing as a modality to prepare preclinical students for clinical clerkships.

This study incorporated medical scribing as an adjunct to our institution’s current preclinical preceptorship program. As in the current mentor-mentee relationship, students witnessed physician-patient interactions and actively participated in SOAP (Subjective, Objective, Assessment, and Plan) note creation and documentation in the electronic medical record (EMR) as a medical scribe. What had been passive observation was replaced with active learning to prepare students for independent EMR documentation in the clinical years. Scribes navigated the electronic medical record, typed new medical terminology, and clarified any emerging questions during the encounter, which is an experience missing by the medical student who is passively observing traditional encounter(s).

The primary aim of this study was to assess student confidence in documentation and clinical skills over time through the use of active scribing activities while also investigating the impact of scribing in medical education on clinical workflow and efficiency. We hypothesized that incorporation of scribing experience into the preclinical curriculum would achieve the following aims: (1) increase student confidence in clinical documentation skills, (2) improve student confidence in navigating the EMR platform, (3) decrease supervising physician documentation time, (4) increase the amount of patient facetime for supervising physicians, and (5) maintain or improve upon current patient satisfaction.

## Methods

This prospective randomized observational trial was approved by the Loyola University Chicago Stritch School of Medicine Institutional Review Board (IRB). All subjects involved in the study signed an IRB approved informed consent form, documenting their willingness to participate in the study. Both participants and evaluators performed this study unblinded. Members of the Loyola University Chicago Stritch School of Medicine Class of 2021 (2nd year medical students) enrolled in the trial. The class included approximately 160 individuals who were invited via email by the senior author (MF) to participate in this study at the beginning of the 2018 academic year. Exclusion criteria was limited to poor academic standing. The study team randomly sorted included students into one of two groups: a control group (non-scribe) or an experimental (scribe) group. The projected endpoint of this prospective feasibility study was the conclusion of one academic year.

All participants had the same outpatient clinic assignment for their preceptorship. Each preceptor was a resident physician who was traditionally paired with two second-year medical students. The non-scribe group participated in the standard second-year preceptorship, which comprises five independent patient encounters throughout one academic year. During a patient encounter, students entered a patient room to obtain a history and perform a brief physical exam. After initial feedback on their patient presentation, the student returned to the room with their preceptor and shadowed as the preceptor conducted their own independent evaluation of the patient. Students ultimately documented the patient encounter on a word processing document and returned with a printed hard copy for review at a later date.

Prior to the start of the study, co-author (VP) provided training materials and sessions for the participants assigned to the scribe group. The training period consisted of three hours of self-directed online learning via powerpoint slides and handouts developed by the co-author (VP) who has had three years of scribing experience and has held positions as a scribe trainer, scribe quality assurance specialist, and chief scribe. The powerpoint slides included an introduction to their role as a scribe and responsibility of creating a medico-legal document for the patient encounter for which they would receive immediate feedback. In addition, VP provided handouts on step-by-step instructions on how to create a note in the EMR, copy or edit smart phrases, and appropriately abbreviate common medical terms. Altogether, the training program exposed the medical student to 4 h of instruction divided between powerpoint slides, handouts and optional in-person sessions for any clarifications.

Throughout the trial, the study groups participated in five patient encounters, procuring a history and performing a brief physical exam. Only upon returning to the room does the non-scribe and scribe encounter differ. While non-scribe students observed (shadowed) the encounter, scribe students documented the encounter in the EMR while the preceptor interviewed the patient. On the same day of the patient visit, the preceptor reviewed the note and provided feedback to the scribe student. For the experience to be a true learning experience, preceptors discussed clinical aspects of the patient encounter, accuracy of their documentation, organization of information within the EMR, and teaching points surrounding clinical decision-making skills with students (both scribe and non-scribe). Specific aspects of feedback sessions were not recorded throughout the study. The supervising physician included an attestation, stating the note was documented with the help of a scribe. Non-scribe and scribe students were never simultaneously participating in the same patient encounter.

Study participants completed a survey, containing thirteen items (designed by authors EM and EW), at the beginning, middle, and end of the study. The students participating in the study self-assessed their own confidence in navigating the EMR and documentation skills throughout the year, both on a Likert scale 1–5. After each encounter, resident physicians also captured both the true time spent documenting (or editing scribe notes) and the patient visit’s complexity (follow-up vs new patient visit). The study team utilized REDCap electronic data capture tools, hosted by the institution, for digital management of survey responses [10, 11].

Furthermore, the authors analyzed patient satisfaction for those patients who were seen by the non-scribe and scribe students. The study participants provided patient satisfaction surveys at the end of their encounter. The authors analyzed the survey results to gauge impact on patient satisfaction and patient encounter integrity over the course of this study. An official translated version of the survey was available for Spanish-speaking patients.

### Statistical methods

This study capitalized on a census of 36 students eligible to participate during the study period, and therefore no a-priori sample size was estimated. We utilized a 1:1 allocation scheme to randomize students to the control (non-scribing) or experimental (scribing) condition [12]. Participant characteristics were reported as valid counts with proportions by assignment. Regarding the primary aim, students rated their confidence in documenting a history, documenting a physical examination, documenting a treatment plan, and in looking up laboratory results, radiology studies, and pathology results after every encounter on an ordinal scale ranging from strongly disagree that they are confident to strongly agree that they are confident. We used multivariable generalized linear mixed-effects models to compare the odds of higher agreement to these survey questions between scribing and non-scribing students while controlling for the number of encounters. In these models, a multinomial distribution with cumulative logit link was specified for the ordinal agreement scale, and the degrees of freedom were estimated using the method of Kenward and Roger [13]. Because students contributed multiple responses of their confidence to the analysis (i.e., 1 per encounter), random intercepts were allowed for each student to account for their correlated responses using a completely general (unstructured) covariance matrix. A similar approach was used to compare the odds of higher patient satisfaction with understanding the doctor and his/her explanation between scribing and non-scribing participants.

Regarding the efficiency of resident physicians, we used a multivariable linear mixed-effects model to estimate the mean difference in resident physicians’ patient facetime and documentation time when supervising scribing versus non-scribing students while controlling for the encounter type (i.e., whether it was a follow-up versus new encounter). Resident physicians contributed this information after every encounter during the study period. As before, random intercepts were allowed for each resident to account for their correlated responses and the degrees of freedom were estimated using the method of Kenward and Roger.

## Results

**Fig. 1:**
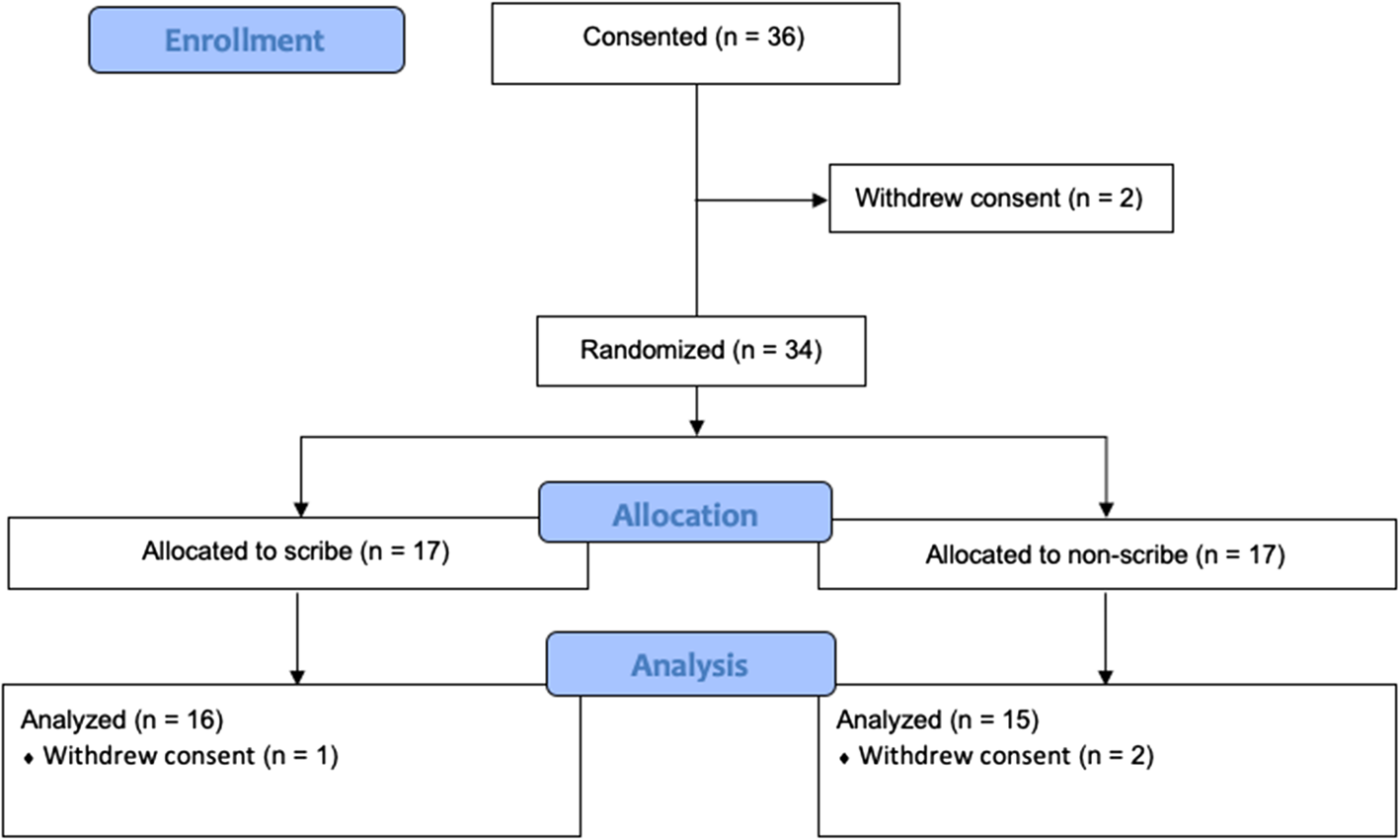
Consort flow diagram of study participants

Figure 1 reports the CONSORT flow diagram of study participants for the trial. Thirty-six participants were consented to participate but two (5.6%) withdrew their consent prior to randomization. Among the remaining 34 students, 17 (50%) were randomized to the control (non-scribe) cohort while 17 (50%) were randomized to the experimental (scribe) cohort. Following randomization, one student (5.9%) in the scribe cohort and two students (11.8%) in the non-scribe cohort withdrew informed consent, leaving 31 students available for the analysis. Key demographics of our study participants are listed in Table 1. Few were fluent in Spanish (*n* = 7/22 or 32%) or had prior documentation experience (*n* = 9/28 or 32%). More than half (*n* = 6/11 or 52%) had prior experience at the clinic site. Less than half (*n* = 13/31 or 42%) had prior EMR experience. The makeup of the scribe and non-scribe groups were not found to be significantly different from one another (*p* > 0.05) based on the various demographic designations.

**Table 1:**
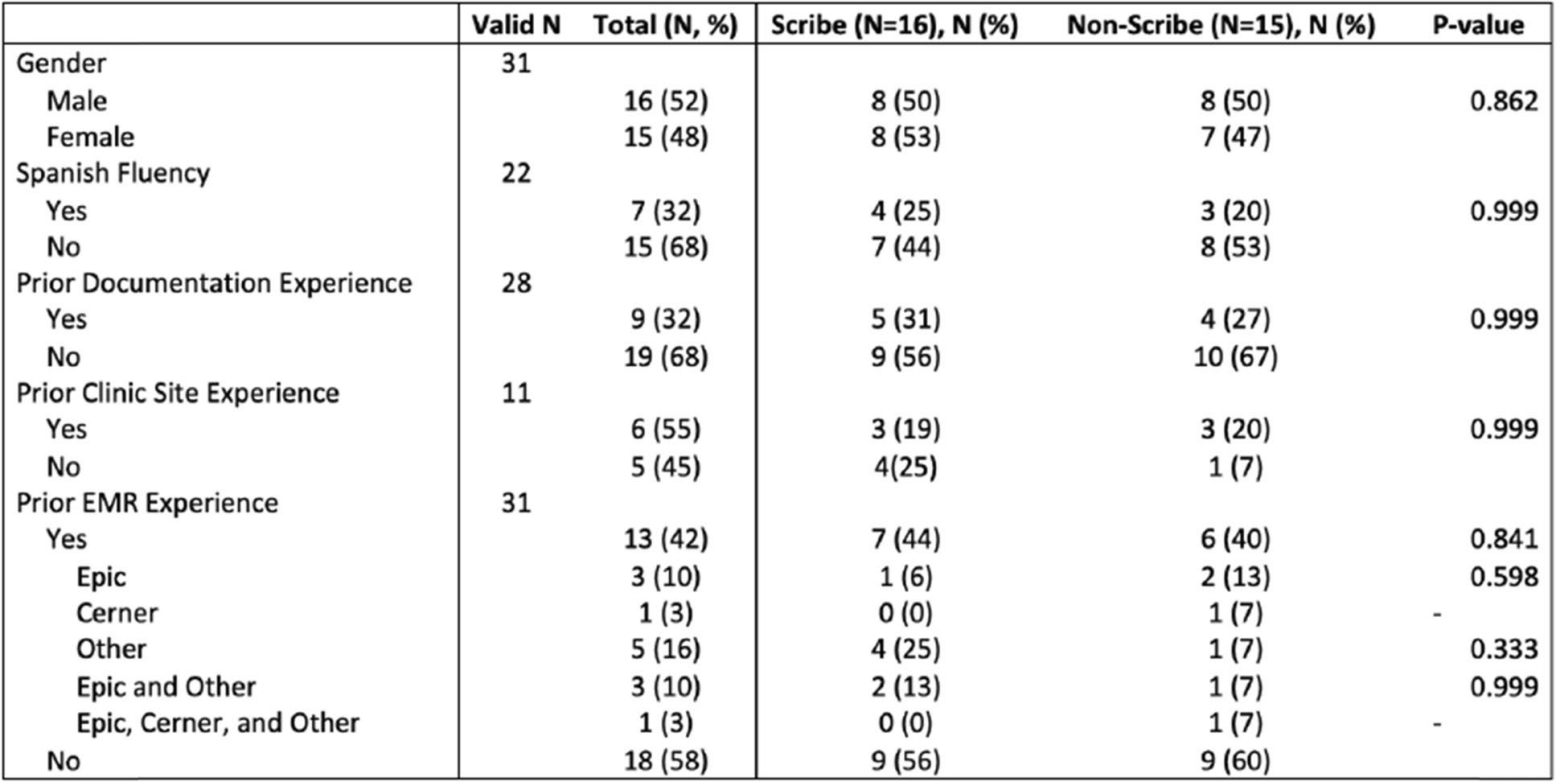
Participant demographics

Table 2 summarizes students’ confidence in documenting aspects of the patient encounter and navigating the EMR. Between scribing versus non-scribing students, there was no significant difference in the odds of reporting higher student confidence for documenting a history (OR = 1.35; *p* = .64), documenting a physical examination (OR = 1.26; *p* = .67), documenting a treatment plan (OR = 1.35;*p* = .50), or looking up laboratory results (OR = 1.64;*p* = .51), radiology studies (OR = 1.54;*p* = .68), or pathology results (OR = 1.26;*p* = .78. However, controlling for scribing status, students were more likely to report higher confidence as they gained experience over the course of the study. That is, for each additional encounter students increased the odds of reporting higher confidence in documenting a history (*OR* = 2.06; *p* < .001), documenting a physical examination (*OR* = 3.14; *p* < .001), documenting a treatment plan (*OR* = 2.46; *p* < .001), looking up laboratory results (*OR* = 1.47; *p* = .001), looking up radiology studies (*OR* = 1.72; *p* < .001), and looking up pathology results (*OR* = 1.69; *p* < .001).

**Table 2:** Odds of higher student confidence

	Valid N	Odds Ratio	***p***
**Documenting History**	31		
Scribe vs non-Scribe		1.35 (0.37–4.93)	.64
Per encounter increase		2.06 (1.57–2.70)	<.001
**Documenting Exam**	31		
Scribe vs non-Scribe		1.26 (0.42–3.74)	.67
Per encounter increase		3.14 (2.31–4.27)	<.001
**Documenting Plan**	31		
Scribe vs non-Scribe		1.35 (0.55–3.31)	.50
Per encounter increase		2.46 (1.91–3.18)	<.001
**Navigating EMR Labs**	31		
Scribe vs non-Scribe		1.64 (0.36–7.49)	.51
Per encounter increase		1.47 (1.19–1.81)	.001
**Navigating EMR Radiology**	31		
Scribe vs non-Scribe		1.54 (0.20–11.97)	.68
Per encounter increase		1.72 (1.34–2.21)	<.001
**Navigating EMR Pathology**	31		
Scribe vs non-Scribe		1.26 (0.23–6.99)	.78
Per encounter increase		1.69 (1.33–2.13)	<.001

*Note*: Valid *N* = The number of students used to compute the estimates. *EMR* = Electronic medical record

**Table 3:** Mean difference in minutes reported by supervising physicians

	Valid N	Mean Difference	***p***
**Facetime between patient and physician**	21		
Scribe vs Non-Scribe		7.29 (−3.34 to 17.91)	.17
Follow-up vs New Encounter		−0.03 (−12.13 to 12.08)	.99
**Documentation Time**	21		
Scribe vs Non-Scribe		−5.75 (−10.64 to −0.87)	.02
Follow-up vs New Encounter		−0.74 (−6.78 to 5.31)	.81

*Note*: Valid *N* = The number of supervising physicians used to compute the estimates

Regarding the efficiency of resident physicians, there was no significant difference in patient facetime (in minutes) when the resident was supervising a scribing versus non-scribing student (*M*_diff_ = 7.29; 95% CI: − 3.34 to 17.91) or when the encounter was a follow-up visit versus new patient visit (*M*_diff_ = − 0.03; 95% CI: − 12.13 to 12.08; *p* = .99). However, controlling for the encounter type (new vs follow-up patient visit), resident physicians spent less time (in minutes) documenting or editing an EMR note when they were supervising a scribing student than when they were supervising a non-scribing student (*M*_diff_ = − 5.75, 95% CI: − 10.64 to − 0.87; *p* = .02). Still, patient satisfaction was unaffected by students’ scribing status. That is, when patients saw a scribing (rather than non-scribing) student, they were 1.07 times more likely to report higher confidence in their doctor’s explanations though this was not statistically significant (95% CI: 0.23 to 5.09; *p* = .93). Conversely, when patients saw a scribing (rather than non-scribing) student they were only 0.93 times as likely to report higher satisfaction with the doctor’s explanation though, as before, this was not statistically significant (95% CI: 0.29–2.92; *p* = .89).

## Discussion

Our pilot trial sought to transform the preexisting second-year preceptorship experience to involve active participation to prepare students for the independent EMR documentation during clinical clerkships. Our study demonstrates that scribing did not negatively impact students’ confidence around clinical documentation and the patient encounter throughout the year consistent with the longstanding goals of the preceptorship program. Moreover, scribing helped reduce resident physicians’ workload while maintaining patient satisfaction.

First and foremost, the pilot studied the hypothesis that exposure to active clinical documentation during preclinical years can accelerate clerkship readiness through increasing student confidence in [1] EMR documentation skills and [2] navigating the EMR for pertinent patient information. Results in Table 2 support the paradigm of shadowing as a mechanism to increase confidence in both documenting the history, physical exam, plan, and facility with accessing and interpreting EMR labs, imaging, and pathology. However, our results do not show a statistically significant difference from the action of scribing itself in increasing either confidence endpoints. This may be due to the small sample size or the fact that students in our shadowing arm were tasked to document the patient encounter “off-site” with eventual feedback from precepting physicians. The participating clinic, resident, and attending supervision limited our enrollment to 36 students. Students were also limited to only 5 patient encounters for scribing activities. Modifications in the future should include expanding clinical sites to reduce student density, allowing for an even greater amount of patient encounters throughout the year. This may allow students to have more opportunities to demonstrate growth and realize self-confidence around the patient encounter. This study is also limited in that other markers for skill acquisition in addition to student confidence were not assessed such as reviewing the students’ navigation of the EMR or peer reviewing their documentation in addition to the preceptor. Additionally, our study is limited in that we did not capture retention of individual medical student scribes’ confidence or clinical skills during the clinical years.

**Table 4:** Odds of a more favorable patient response for scribes versus non-scribes

	Valid N	Odds Ratio	***p***
**Understand Doctor**	29	1.07 (0.23–5.93)	.93
**Health Plan Explanation Satisfactory**	29	0.93 (0.29–2.92)	.89

*Note*: Valid *N* = The number of patients used to compute the estimates

Secondly, the pilot aimed to improve the quality of the encounter through decreased preceptor documentation time while preserving patient satisfaction. Our results demonstrate physicians spending less time documenting with no observed difference in face-to-face time. There was no observed difference in patients’ understanding of the clinical treatment plan, or their satisfaction with explanations provided. These results are in line with prior published research on the benefit of medical scribes [4]. It is worth addressing the real-world significance of reducing documentation time. If one extrapolates an individual encounter with a medical student scribe in our current model to precepting a medical scribe in a half-day or full day clinic, the time savings projects to as little as 30 min and potentially as great as 90 min per supervising physician. This time savings and greater interaction between students and precepting physicians can be further investigated in a future study to ideally be used for more patient care, student education, and even professional development.

These endpoints are particularly important as a documented anecdotal complaint in the medical community remains that the addition of the EMR to a patient encounter detracts from its genuine nature. A recent meta-analysis of 22 studies examining EMR impact on patient perception of face-to-face patient-doctor communication demonstrated no change with EMR use, and even a positive impact in some [5, 14, 15]. Our study supports that meta-analysis and demonstrates that the extra time required to document a visit can be simultaneously integrated into the encounter without impacting patient perception of degree of direct interaction with their physician, or their understanding of the treatment plan.

Nevertheless, this study is the first of its kind to evaluate the utility of medical scribing in preclinical patient encounters. While this study is limited with its small sample size and ability to investigate any downstream value from scribing during clinical rotations, it has the benefits of minimal curricular redesign and implementation. Given the pre-existing curricular restraints, medical students were able to participate in scribing and shadowing encounters (approximately once every other month) throughout the academic year. This interval between encounters may have been a barrier to students’ assessment of their confidence in clinical decision-making and patient encounters. Additionally, our patient population reported high satisfaction scores at baseline (in our study and historically in this clinic), so it is difficult to assess any impact that the SCRIBE intervention had on perception of the patient encounter.

Suggestions for further research include using the results of this pilot trial to power a larger trial that includes different clinical environments with more consistent and regular student scribing immersion throughout the academic year. This would increase the number of patient encounters and both student and resident evaluations. Further investigation on student skill acquisition would also be beneficial such as reviewing the students’ navigation of the EMR after each encounter and peer reviewing their documentation in addition to the evaluation completed by the preceptor. It would further benefit leaders in medical curricula design to investigate whether preclinical scribing leads to long-term benefits during the clinical years. Are students more familiar with our EMR system able to better focus on patient care or self-directed learning during their rotations?

## Conclusion

This pilot demonstrated that scribing is not associated with significantly increasing student confidence for clinical documentation or EMR navigation skills. However, our results show that preclinical scribing could improve the quality of the encounter through [1] decreased supervising physician documentation time without [2] decreasing patient satisfaction..

With the emphasis in medical education changing to prioritize clinical learning, early integration of scribing could potentially bridge preclinical and clinical education regarding documentation while reducing current burdens on supervising faculty. Future studies investigating the use of time saved by reduced documentation burden and the relationship between scribing and objective skills acquisition beyond self-perception of readiness may further strengthen the argument for institutions to adopt preclinical scribing in medical curricula.

## Data Availability

The datasets used and/or analyzed during the current study are available from the corresponding author on reasonable request.
